# Do health sector measures of violence against women at different levels of severity correlate? Evidence from Brazil

**DOI:** 10.1186/s12905-022-01813-y

**Published:** 2022-06-13

**Authors:** Sarah Anne Reynolds

**Affiliations:** School of Public Health, University of California, Berkeley, USA

**Keywords:** Intimate partner violence, Hospitalizations, Homicides, Aggression, Mandatory reporting, Brazil

## Abstract

**Objective:**

To evaluate if characteristics of reports of violence against women at different levels of severity are similar and to test if their prevalence is correlated at the municipal level.

**Methods:**

I use data from women ages 15–49 who were killed by homicide in Brazil’s national death registry (N = 14,373), were hospitalized for aggression (N = 14,701), or were included in the medical mandatory reports of incidents of violence against women (N = 42,134) between 2011 and 2016 in select municipalities. I provide national level descriptive statistics from 2016 contrasting distributions of victims (age, education, and race) and distributions of the characteristics of the incidents (location and time of day). Then, for 63 municipalities with a high number of violent incidents, I calculate the correlation coefficients between measures of violence against women using quarterly data from 2011 to 2016. I use multiple regression of municipal characteristics at baseline to examine which factors (poverty, spending, health, and civic engagement) predict the correlation.

**Results:**

Victim characteristics and incident characteristics are similar across the measures of violence at the national level. Despite these aggregate similarities, correlations at the municipal level are quite varied, ranging from − 0.69 to 0.83. I find no municipal characteristics that consistently predict these correlation coefficients.

**Conclusions:**

Despite some similarities at an aggregate level, these measures of violence against women do not have consistent patterns of correlation at the municipality level. Measures of severe levels of violence against women are not good proxies for incidence of violence at less severe physical levels. Lack of correlations could be due to differences in reporting, but may also be due to differences in underlying processes that share similar victims and event characteristics.

## Introduction

Intimate partner violence (IPV) can occur at varying levels of physical severity: from a slap to homicide, all are classified as intimate partner violence. Measuring violence against women is difficult due to the fact that much violence occurs in the home. However, if the aggression is severe enough physically, these incidents can be documented within the health care system. Few health reports indicate the perpetrator, but since the most common perpetrators of violence against women are male intimate partners or ex-partners, these classification measures for violent cause of injury can be a good proxy for IPV, even though the relationship is not specified [1]. Three administrative measures that could be considered to be on different portions of this spectrum are reports by health care workers of violence against women, hospitalizations for aggression, and female homicides. Homicide clearly is the most severe, but the other measures have not been explicitly classified. Nevertheless, the Conflict Tactic Scales, a survey measure including physical intimate partner violence, has been shown to accurately map items along a single dimension of physical violence continuum [2], often divided into less severe and more severe [3]. Thus such an extrapolation to physical IPV in administrative data is plausible. Yet the similarities and correlations of these measures have not been systematically explored. If these measures move similarly, this suggests they are capturing some of the same behavior manifesting at different levels of severity. On the other hand, uncorrelated movement across measures suggests these reports are capturing different phenomena.

Research has shown distinct subtypes of IPV exist [4], though it may result in some of the same physical violence. Characterological IPV is when the perpetrator (typically male) uses violence as a means of inducing fear and controlling the victim; Situational IPV is mutual, low-level violence (i.e. pushing or grabbing) perpetrated by both partners as a means of conflict management [5]. Straus and Gelles found that 50% of physically aggressive couples exhibit low-levels of mutual violence that is situational in nature (1986, reported by Friend et. al.) [5, 6]. On a survey of low-level violence in the United States, both women and men report similar levels of violence, suggesting situational violence is prevalent for both genders; on the other hand, in shelters and court cases, which deal with the most severe cases, characterological cases of violence against women dominate [7]. In Brazil, in contrast, violence against women has been conceptualized as being on a continuum, with psychological, sexual, and physical violence potentially culminating in death; the chronic nature of patriarchal dominance suggests an underlying cause of all types of violence [8]. Yet, as far as I am aware, research using administrative health data has not confirmed incidents of violence at different levels of severity emerge from distinct subtypes of IPV. Data on perpetrator type is not typically available, but if patterns exist across the different severity levels, it may suggest the underlying violence springs from a similar mentality. Furthermore, if movement in measures of violence across levels of severity is similar, indicators of violence at one level could be a plausible proxy for indicators at a distinct level of severity.

Brazil is an excellent location for this type of study for several reasons. First, the health system collects administrative data on violence at multiple levels: reports by health care workers in all health care facilities, hospitalizations, and homicides. Secondly, in spite of having comprehensive legislation against violence against women [9] and in spite of being in the top quartile of Latin American countries [10] with the strongest attitudes against violence against women, Brazil suffers high levels of violence against women, including having one of the highest female homicide rates in the world [11]. The high rates indicate non-negligible incidence allow for meaningful comparison across severity levels of violence against women. Previous research suggests that though the legislation protecting women is strong, the police force is not always trusted to do so [9]; thus, the use of health data rather than criminal data is more likely to reflect reality as there will be fewer reporting concerns.

Previous research on correlations of measures of violence against women in Brazil have generally focused on examining predictors of one type of outcome. For example, Meneghel et al. find that poverty, male aggression, and conservative religion are factors most strongly associated with female homicides in capitals and large Brazilian cities [12]. This research contributes to the literature to evaluate if characteristics of reports of violence against women at different levels of severity are similar and to test if their prevalence is correlated at the municipal level; if so, this would allow conclusions such as those in Meneghel et al. to be extrapolated to different levels of violence. In an additional study, Barufaldi et al. find that the women for whom health care workers have indicated as victims of violence are more likely to appear in the homicide records that women who do not appear in this registry [13]. The current study contributes in assessing if such conclusions could hold at an aggregate level: do municipalities with high rates of reports of violence against women also have high rates of female homicide?

## Methods

### Data sources

Health data on violence against women come from three sources. For each source, I consider only females ages 15–49.

Vital statistics data on homicides are from Brazil’s National mortality database Sistema de Informações de Mortalidade (SIM—System of Mortality Information) [14], a registry of every death in the nation. I consider a death to be a homicide if it is coded to be assault (i.e., codes ICD-10 X85-Y09) or if it was categorized as a homicide. The discrepancy between these definitions is only 6%.

The Sistema de Comunicação de Informação de Hospitalar e Ambulatorial (CIHA—System of Communication of Hospital and Outpatient Information) [15] includes information from public and private hospitals. Hospitalizations are recorded when the physician fills out a form called Autorização de Internação Hospitalar (AIH—Authorization for Hospitalization), which is used to reimburse the hospital for the procedures performed on its patients. This data only captures the most severe incidents because, according to the Ministry of Health, this form is filled out when patients need to be admitted overnight. I exclude hospitalizations that resulted in deaths, as these would be duplicates with the SIM death registry. The reason for intake is classified with the ICD-10, which I use to select the hospitalizations for assault using the same codes as in the homicide data (X85-Y09). Though the primary cause typically indicates the location of the injury (e.g. trauma of the knee or leg), the secondary cause often suggests the reason for the injury (e.g. accident, assault).

There are two concerns regarding hospitalization data accuracy. The AIH form is also filled out when patients need a procedure, so some non-overnight patients may also be in the system. However, few of those procedures are associated with assault and thus are unlikely to bias our results. The other concern is that hospitals cannot report more patients than the number of beds they have registered at the centralized health care system. Thus, the system may be under-reporting incidents in crowded hospitals.

The Sistema de Informação de Agravos de Notificação (SINAN—Notifiable Diseases Information System) are reports filled out at the health care units by the attending medical personnel (e.g. doctor, nurse, dentist, psychologist, social worker). These reports are mandatory for cases of domestic violence (including IPV) and violence against women, children, and elderly [16]. However, the follow-through of contacting police or social workers, for example, is only required for children and elderly, though implementation of federal law locally can vary widely across the country. Although psychological, financial, and sexual violence can be reported, I only include SINAN cases for which sexual and physical violence was reported, since these correspond to the assault categories of the ICD-10 codes used in the homicide and hospitalization data. I divided the SINAN data into less severe and more severe reports. The more severe reports were those from hospitals and emergency rooms while the less severe were those reported by clinics and other establishments. I also considered the subset of data in which the perpetrator was identified as partner or ex-partner; I add the qualifier “IPV” to this subsample. This subsample includes both clinic reports and hospital reports to maintain a larger sample size.

There may be some noise in how incidents were classified. Depending on availability of clinics or the hours they were open, some women may have used hospital emergency rooms for less severe incidents. Similarly, clinics may refer women with more severe injuries to hospitals. Unfortunately, this information is unknown in the data and has to be considered noise for the purposes of this analysis. While the overnight hospitalizations (and perhaps even a few deaths) may appear in the SINAN reports of hospitalizations, the magnitude difference is so large that the overnight hospitalizations would only be a very small part of the noise in the SINAN reports from hospitals. Even if reported in the SINAN reports, women who were hospitalized overnight for aggression or were murdered should still appear in the CIHA and SIM registries as these are registries are for different purposes than the SINAN reports. Additionally, I excluded any overnight hospitalizations that resulted in death from that categorization, so there should be minimal noise in that regard to overlap between the overnight hospitalization and homicide categories.

Several municipal measures from 2011 relating to policy that may influence IPV or IPV reporting are used in the analysis: a poverty measure, if the municipality has a woman’s police station, if the municipality has a local civil police station which investigates other crimes (as opposed to another municipality being in charge of investigations), police investment, health investment, civic engagement, the share of population that is female, and population size. For a number of these measures, I used exploratory factor analysis to create an index of several correlated variables. A poverty measure was created from measures of Bolsa Familia coverage (portion of eligible women) and Bolsa Familia transfer per woman from data from the Ministerio de Desenvolvimento Social (MDS—Ministry of Social Development) [17], combined with the human development index [18]. Using data from the Brazilian Treasury (Finanças do Brasil—FINBRA) [19, 20], a police investment index was created from per capita spending on policing and per capita spending on public safety. A health investment index was created from per capita spending on clinics, per capita spending on health establishments, per capita spending on hospital beds, per capita health spending in general, per capita spending on hospital aid, and per capita spending on social assistance. Finally, from the Survey of Basic Municipal Information (MUNIC) [21], a civic engagement measure was created from variables indicating if the municipality had a safety council, a health council and a human rights council.

### Sample size and definition

For the correlation analysis, I use quarterly observations, for a total of 24 periods. I select cities that have a high volume of incidents reported to DataSus: I only used municipalities in which all outcomes had observations in at least half of the quarters. This restriction reduces inflated correlation due to zeros being correlated with zeros. In this longitudinal analysis, I limit SINAN reports to facilities that were already reporting in 2011—the first year the system was nationally required—to avoid bias due to an increase in reporting rather than a change in incidence. (This approach has been used elsewhere [13]). Brasilia, the federal capital, was excluded due to having more national level influences than other municipalities and I also eliminated two municipalities that were missing data on poverty, resulting in a total of 63 municipalities. Though these 63 municipalities are only about 1% of the municipalities reporting violence, they include 30% of the population of women ages 15–49 and around a fifth of the incidents.

### Analysis

I contrast distributions of victims in the different reporting systems by age, race, and education. Education data is not available for hospitalizations. For reports and homicides, I also examine time of day of the incident and if the incident occurred at home or not. For the reports, additional information was available on if these incidents were single events or multiple events, if a weapon was involved, and the identity of the perpetrator. Weapons were broadly defined and could include household objects or sticks, for example, in addition to knives and firearms.

I test three hypotheses. First, that violence against women is correlated at different levels of severity. I aggregate the data to get a count of the incidence in each municipality for each quarter from the period 2011–2016. Then, for each municipality, I calculate the correlation of the quarterly incidents: reports from clinics, reports from emergency rooms and hospitals, overnight hospitalizations, and homicides. I use a t-test to determine if the average correlation between each of these groups is distinct from zero. Using the same method, I test the hypothesis that IPV reports are associated with hospitalizations and homicides. Finally, I test the hypothesis that municipal characteristics predict heterogeneity in the correlations. I use OLS multiple regression to examine if municipal characteristics explain why within some municipalities’ measures of violence are more correlated than in others.

## Results

From the 2011–2016 panel of the 63 municipalities used in the correlation analysis, I present descriptive statistics on characteristics of victims and incidents using the full dataset from all registries of violent incidents as well as for the subset of IPV incidents from the SINAN reports; I also present the same descriptive statistics at a national level for 2016 (Table 1). I find that though each data source has different levels of missing information regarding traits of the victims and the incidents, overall, there are similarities in these characteristics for all levels of violence (Table 1). This is true for the incidents in the correlation sample from 2011 to 2016 as well as for the full universe of victims and incidents in 2016. Additionally, there were very few differences in the distributions that were larger than 5% when contrasting 2016 data with the correlation sample. Exceptions were that 2016 had more information on education in the reports, and the larger municipalities (correlation sample) had fewer homicides occurring at home. Yet the multiple incidents were higher in the correlation sample (which comprises more populated municipalities) which may reflect that urban women can seek medical attention more frequently. The percentage of IPV incidents in the larger municipalities was smaller than the percentage in all of Brazil, suggesting urban crime may represent a number of these incidents.

**Table 1:** Characteristics of victims and incidents of violence against women ages 15–49 in the Brazilian health care system. *Sources*: Brazil's DataSus registries: SINAN, CIHA, SIM

Level of severity Source	2011–2016, correlation sample of 63 large municipalities				2016, all incidents in Brazil			
	Clinic rpts	Hospital rpts	Overnight hosp.	Homicides	Clinic rpts	Hospital rpts	Overnight hosp.	Homicides
	SINAN	SINAN	CIHA	SIM	SINAN	SINAN	CIHA	SIM
Mean age (between 15 and 49)	29.4	27.9	30.6	31.1	29.3	28.6	30.4	31.6
Standard deviation	9.0	9.1	9.4	9.8	9.3	9.2	9.3	9.8
Race available (%)	84.1	80.9	49.2	98.3	92.5	85.4	63.4	96.9
White	49.8	40.2	34.3	38.9	45.3	41.6	31.7	37.2
Non-white	50.2	59.8	65.7	61.1	54.7	58.4	68.3	62.8
Education available (%)*	70.3	50.9	N/A	92.6	73.0	56.4	N/A	92.9
Illiterate	5.6	5.7		1.5	8.2	6.3		0
Lower primary	24.1	25.9		1.8	26.4	26.1		3.4
Upper primary	29.5	29.5		11.5	29.8	30.2		12.9
Secondary	34.0	32.8		32.1	29.5	31.6		31.5
Tertiary	5.6	4.7		30.7	4.0	4.6		29
Time of day available (%)	59.4	59.1	N/A	65.3	69.1	64.0	N/A	76.5
Morning	19.6	16.6		25.1	17.7	15.4		26.7
Afternoon	25.2	22.0		23.7	25.4	22.6		24.9
Night	55.2	61.4		51.2	57.0	62.1		48.4
Location information available (%)**	92.0	79.0	N/A	59.9	95.6	82.5	N/A	67.1
Occurred at home	73.0	57.2		41.6	72.4	61.1		47.0
Occurred elsewhere	27.0	42.8		58.1	27.6	38.9		52.1
Multiple incident info (%)	79.1	66.7	N/A	N/A	87.1	71.7	N/A	N/A
Mutiple incidents	63.6	42.9			56.0	41.1		
Single incident	36.4	57.1			44.0	58.9		
Source of injury known (%)	93.0	89.6	N/A	N/A	93.0	91.3	N/A	N/A
No weapon	83.1	73.7			83.1	77.0		
Weapon	16.9	26.3			16.9	23.0		
Perpetrator relationship identified (%)	94.1	83.7	N/A	N/A	96.3	86.2	N/A	N/A
Partner	44.5	28.5			44.7	33.8		
Ex-partner	19.1	12.9			16.6	13.8		
Stranger	8.6	22.1			9.2	18.4		
Other relationship	27.8	36.5			29.5	34.0		
# Of incidents (women, ages 15–49)***	5280	9116	2450	2396	22,669	43,719	5496	7164
Percent of 2016 total/ # of municipalities reporting incidents	23.29%	20.85%	44.58%	33.44%	2370	2942	1272	2183

Excludes self-inflicted (reports) and suicides (homicides)

The Correlation Analytical Sample is observations from municipalities with fewer than 12 quarters of data missing between 2011 and 2016

The Correlation Analytical Sample only includes SINAN data from facilities reporting since 2011

*Education levels for reports are completed levels; for homicides they are levels initiated or completed

**For homicides, death occurred at hospital indicates location of aggression unknown

***Yearly average for correlation sample

On average, victims are around 30 years old, and are more likely to be non-white than white (Table 1). In Brazil, however, just under half of the population is white, suggesting disproportionate victimization of people of color. In addition, less educated women are also more represented as victims than those with secondary school or less, even though more than 60% of Brazilian women have completed secondary school; among younger women this statistic is even higher as the current enrollment of girls in high school is close to 100% [22].

Both incidents reported in the violence registry and homicides occurred more frequently at night than in the morning or afternoon, and violence reports showed incidents more likely to occur at home than elsewhere (Table 1). The homicide data indicated that deaths at home were slightly less likely than elsewhere, but for a large portion of homicides the location of death was indicated to be the hospital, thus the location of the incident itself was unknown.

**Fig. 1:**
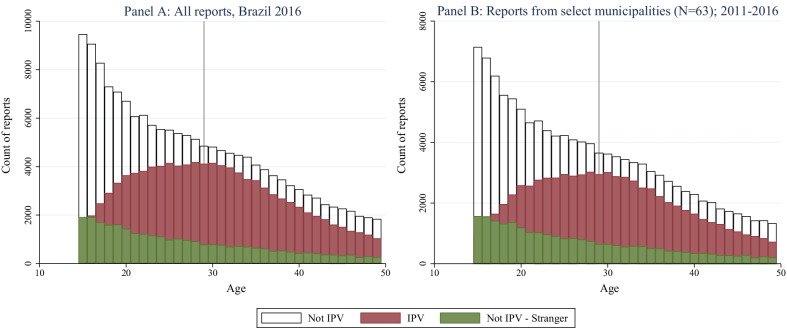
Reports of violence by perpetrator type for Brazil and select municipalities. *Source*: SINAN. *Notes*: A report of violence is characterized as a SINAN report in which physical and/or sexual violence was reported. We use information on the perpetrator of the violence that generated the mandatory report to infer whether or not it is a case of intimate partner violence. We classify a report as being related to intimate partner violence when the perpetrator is identified to be either a partner or former partner

In the violence registry reports, almost 90% identified the relationship of the perpetrator to the victim (Table 1). Less than 15% of perpetrators were unknown to the victim, with around half of these incidents being due to intimate partner violence, perpetrated by the partner or ex-partner of the victim. Figure 1 illustrates how the relationship changes with age: older women are more likely to be victims of IPV, rather than victims of violence from other known persons with whom they are not partnered. Additionally, about half of the registry reports indicated that the violence was recurring. However, I am unable to determine if re-occurrences were also present in the data since this data was de-identified and unlinked across victims. Characteristics of victims and incidents of IPV were similar for 2016 and for the correlation sample (Table 2).

**Table 2:** Characteristics of victims and incidents of IPV. *Sources*: Brazil's DataSus registries: SINAN

Level of severity Source	2011–2016, correlation sample		2016, all incidents	
	IPV reports	All reports	IPV reports	All reports
	SINAN	SINAN	SINAN	SINAN
Mean age (between 15 and 49)	30.0	28.6	30.3	29.0
Standard deviation	8.4	9.1	8.6	9.2
Race available (%)	85.9	77.8	90.8	86.6
White	44.4	42.7	44.0	43.4
Non-white	55.6	57.3	56.0	56.6
Education available (%)*	63.6	55.5	67.3	61.5
Illiterate	6.4	5.7	7.7	6.8
Lower primary	25.6	24.9	26.9	25.7
Upper primary	28.1	29.4	28.5	29.8
Secondary	34.5	33.8	31.5	31.8
Tertiary	4.5	5.0	4.1	4.5
Time of day available (%)	64.9	61.2	68.3	65.4
Morning	17.8	17.5	16.0	16.3
Afternoon	21.5	23.1	21.7	23.4
Night	60.7	59.3	62.3	60.2
Location information available (%)**	92.7	81.2	94.0	86.7
Occurred at home	80.3	63.6	81.7	65.6
Occurred elsewhere	19.7	36.4	18.3	34.4
Multiple incident info (%)	81.3	69.0	83.7	76.4
Mutiple incidents	72.3	51.9	66.8	48.4
Single incident	27.7	48.1	33.2	51.6
Source of injury known (%)	95.5	91.3	95.6	92.0
No weapon	82.7	76.6	82.6	78.9
Weapon	17.3	23.4	17.4	21.1
Perpetrator relationship identified (%)				
Partner		35.8		37.9
Ex-partner		15.4		15.7
Stranger		15.8		14.5
Other relationship		33.0		31.9
# Of incidents (women, ages 15–49)***	7022	19,467	42,205	86,861
Percent of 2016 total/# of municipalities reporting incidents	17%	22%	3113	3944

Excludes self-inflicted (reports) and suicides (homicides)

The Correlation Analytical Sample is observations from municipalities with fewer than 12 quarters of data missing between 2011 and 2016

*Education levels for reports are completed levels; for homicides they are levels initiated or completed

**For homicides, death occurred at hospital indicates location of aggression unknown

***Yearly average for correlation sample

Table 3 contrasts characteristics of the 63 municipalities which are in the correlation sample with other Brazilian municipalities. These municipalities are much more populous than the other municipalities and most have women’s police stations, in contrast to municipalities not included in the sample. Additionally, the largest difference in rates is in hospitalizations, which may be because the correlation sample is comprised of larger municipalities with more hospitals and more hospital beds.

**Table 3:** Municipal summary statistics, select municipalities, Brazil 2011–2016. *Sources*: Brazil's DataSus registries: SINAN, CIHA, SIM

Variable	Correlation sample	Other municipalities
	Mean (standard deviation)	Mean (standard deviation)
Report rate (clinics)	5.96	6.50
	(2.02)	(0.50)
Report rate (hospitals)	17.70	8.98
	(2.83)	(0.49)
Hospitalization for aggression rate	3.34	2.00
	(0.43)	(0.16)
Female homicide rate	1.64	1.53
	(0.16)	(0.10)
Poverty index	− 1.00	0.01
	(0.05)	(0.01)
Women's police station	0.90	0.06
	(0.04)	(0.00)
Civil police station	0.81	0.15
	(0.05)	(0.00)
Police spending	0.71	− 0.01
	(0.17)	(0.01)
Public health spending	− 0.12	0.00
	(0.03)	(0.01)
Civic engagement index	0.44	− 0.01
	(0.05)	(0.00)
Share female population	0.51	0.49
	(0.00)	(0.00)
Female population ages 15–49	259,510	7238
	(58,468.18)	(310.86)
N	63	5502

**Fig. 2:**
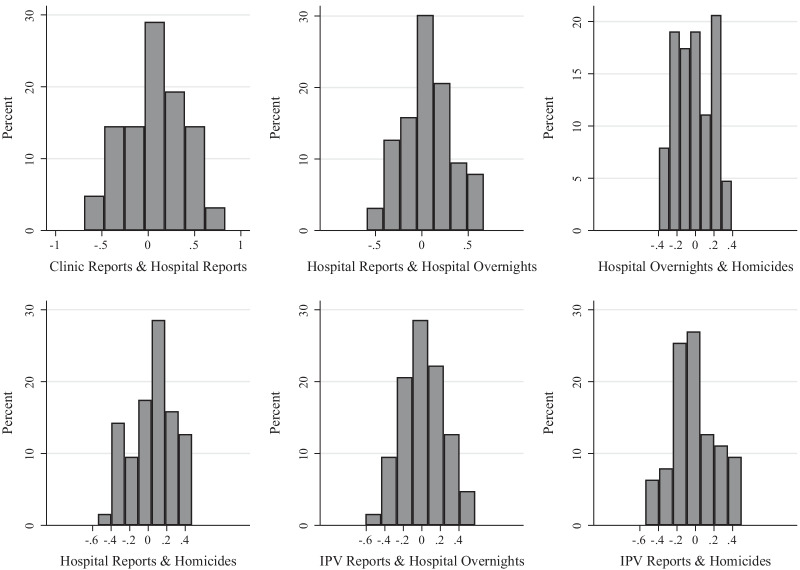
Within-Municipality Correlations of Measures of Violence Against Women; Select Brazilian Muncipalities, 2011–2016. *Source*: Brazil’s DataSus SIM, CIHA & SINAN, women ages 15–49. N: 63 municipalities with quarterly observations from 2011 to 2016

When I calculated the correlation between the various types of violence for each municipality between 2011 and 2016, all within-city correlations of violence are generally evenly distributed around zero (Fig. 2). The correlations ranged from − 0.692 to 0.831. Both of these extreme values were for the within-municipality correlations of registry reports from clinics and hospitals. The two violence measures with the narrowest range of correlation coefficients were overnight hospitalizations and homicides, ranging from − 0.395 to 0.392.

When I regressed each municipality’s correlation coefficient on municipality characteristics, I found very few factors that were associated with the correlation being more positive (Table 4). Over half the municipal characteristics never emerged as significant, and half of the regressions had no significant associations between correlations of levels of violence and municipal characteristics. Thus, I caution that the few significant results that were found, described in the next two paragraphs, may be spurious.

**Table 4:** Muncipal characteristics do not predict correlation between measures of violence against women in the Brazilian health care system

Correlations between these measures	Clinics and hosptializations (SINAN Reports)		Hospitalization (SINAN reports) and overnight hospitalizations		Hospitalizations (registry) and homicides		Overnight hospitalizations and homicides		IPV (registry) and overnight hospitalizations		IPV (registry) and homicides	
	Coef	SE	Coef	SE	Coef	SE	Coef	SE	Coef	SE	Coef	SE
Poverty index	0.11	(0.15)	0.11	(0.11)	0.06	(0.09)	0.14	(0.10)	0.02	(0.09)	0.08	(0.11)
Women's police station	− 0.14	(0.16)	− 0.14	(0.12)	0.03	(0.10)	− 0.09	(0.11)	− 0.12	(0.10)	0.04	(0.11)
Local police station	− 0.1	(0.12)	− 0.12	(0.09)	0	(0.07)	0	(0.08)	0.11	(0.08)	0.03	(0.09)
Police spending	0.03	(0.04)	− 0.02	(0.03)	− 0.02	(0.02)	0	(0.03)	− 0.02	(0.02)	0.01	(0.03)
Public health spending	0.12	(0.26)	0.32	(0.20)	0.23	(0.16)	0.48***	(0.17)	− 0.18	(0.16)	0.15	(0.19)
Civic engagement index	− 0.2	(0.12)	0	(0.09)	− 0.01	(0.07)	0.06	(0.08)	− 0.04	(0.07)	0.05	(0.08)
Share of population female	− 10.32*	(6.01)	5.56	(4.54)	3.89	(3.58)	0.9	(3.98)	3.56	(3.74)	2.56	(4.24)
Log female population 15–49	0.13*	(0.07)	0	(0.05)	0.01	(0.04)	0.04	(0.04)	− 0.10**	(0.04)	− 0.02	(0.05)
Constant	4.09	(2.77)	− 2.32	(2.12)	− 2.02	(1.67)	− 0.63	(1.86)	− 0.58	(1.74)	− 1.03	(1.98)

*p < 0.1, **p < 0.05, ***p < 0.01

N = 63 municipalities

2011–2016, correlations of quarterly data

Log female population was the most significant predictor: it was positively associated with the correlation coefficient between reports from clinic and hospital registries but negatively associated with correlation coefficients between IPV and overnight hospitalizations. The positive correlation would be expected as larger municipalities would have the potential to have greater counts of both outcomes, so there may be fewer counts of zero. A justification for the negative correlation between IPV from registries and overnight hospitalizations is that in larger municipalities there are more clinics to treat less severe wounds, so these do not become more severe avoid being treated in the hospital.

The share of female population was also negatively associated with the correlation coefficient between violence reported by clinics and by hospitals in the SINAN registry. This suggests that when there are fewer women relative to men, the lesser and more severe violence incidents move similarly, perhaps because men must treat women consistently. Finally, there was a positive association between public health spending and the correlation between overnight hospitalizations and homicides. This is expected, as more public health spending per capita might prevent both hospitalizations and homicides, allowing these types of violence to have similar trends.

However, as mentioned earlier, these patterns do not repeat for different outcomes of a similar relationship. For example, we might expect patterns in the correlations between clinics and hospital reports to echo patterns in correlations between overnight hospitalizations and homicides. Thus, I re-emphasize that the correlations found may be spurious.

## Discussion

This study contrasts reports of violence against women at different levels of severity found in the health system in Brazil. There are many similarities between characteristics of victims of violence against women of different levels of severity. The victims are more likely to be black and have lower levels of education than the average Brazilian woman, indicating that marginalized populations are more at risk of suffering violence against women, as has been generally found globally [1, 23–26] and prior in Brazil [11]. Women within these marginalized populations often are more economically dependent on their partners than more privileged women. A combination of low earning potential, lack of childcare, and possibly family support stretched thin implies that women have fewer options outside the partnership and thus suffer more abuses than women who are more able to separate [27, 28]. Additionally, marginalized women may live in areas less-served by the government and thus institutions may not reach them such as women’s police stations, which have been shown to reduce female homicides in Brazil [29]. Recent research indicates black and brown women receive differential treatment at some existing police stations [30].

Among the less severe levels of violence, intimate partner violence is more common in clinic registries than in hospitalization registries;hospitalizations include more conflicts with strangers and non-family members. I speculate this is the case because conflicts with strangers and non-family are not often physical. Thus when these conflicts are physical, the results are more severe since the type of people to engage in physical altercations with non-family already are disregarding social protocol. Never-the-less, IPV still accounts for around 50% of the incidents, with strangers only accounting for about a fifth of the SINAN incidents, as Waiselfisz has also documented [11]. Although the perpetrator is unknown for overnight hospitalizations for aggression and homicides, because the characteristics of victims and events were quite similar, it is a plausible that a similar portion are due to IPV. Incidents were most likely to occur at night and at home, suggesting that typical daytime hours of police stations are not optimized to protect against this type of violence [31]. Additionally, partners interact most with each other at home and the timing of a conflict is more likely to be at night when both are less likely to be working.

Findings suggest policy makers cannot assume that violence against women has similar timing patterns at different scales of violence, even though overall descriptive statistics are similar. This indicates a need for measurement of violence at all levels, and that one cannot assume the extreme violence measures are good proxies for violence at less severe levels. This suggests that valuable studies such as that of Meneghel et al., which examines which municipal characteristics are correlated with feminicides, should be replicated for reports of violence against women from health workers and for hospitalizations [12]. Although the null result found in this study could be from noisy data, it may indicate different underlying violence mechanisms behind the different types of violence: situational violence and characterological violence may have distinct underlying triggers. For example, stress may result in more situational conflict while characterological violence may be more consistent in nature.

It is important to note that administrative data at a national level naturally has disadvantages. Brazil is an enormous nation with heterogeneity in implementation of federal laws throughout the country. Similarly, there are differences in quality of health care and policing across regions. For example, different states and municipalities have different local laws regarding protocols for how police notification must occur. Yet because the correlations are within-municipality correlations, it is plausible that these factors similarly impact the different level of violence reports in similar ways. Future research could test this hypothesis further.

There are several limitations to this study. The outcomes are low-frequency events. While of higher incidence than the homicides and overnight hospitalizations, even the reports are artificially low for the correlation analysis because they were only sourced from facilities that were already reporting in 2011 to avoid noise from an increase in reporting rather than an increase in events. While these reports are not subject to reporting bias by the patient, the health worker may underreport by failing in awareness or time to report. I also limit our municipalities to the ones with the most reported incidents, so correlation results are not representative for all of Brazil. I cannot distinguish between repeated victimization of the same woman and the data has some noise, in that the same incident could appear in multiple sources.

Nevertheless, this study has a number of strengths. Violence severity has not yet been examined at as many levels before using administrative data, as far as I am aware, and analyses are cross-sectional (examining characteristics of victims and incidents) and longitudinal (examining if movement in violence at different levels of severity are correlated). The mortality and hospitalization data is well-established and generally considered reliable, while the novel data from SINAN is also considered good quality [32, 33]. The focus on municipalities with the highest number of incidents includes a large portion of Brazilian women and allows for meaningful correlations.

Like Brazil, many countries already monitor homicides and hospitalizations using IDC-10 codes. However, unlike Brazil, less severe levels of violence are not yet widely monitored elsewhere. The lack of correlation across levels of violence suggests that more monitoring is needed at lower levels of severity. Health registries documenting incidents of violence may be useful for such monitoring, and Brazil provides an excellent example of how a registry such as SINAN can be implemented. Because mandatory reporting of violent incidents against women to legal authorities is questionable, using a reporting system for such purposes should be carefully evaluated [34]. Even without linkages to the legal system, the reports, as in the Brazilian case, would be used for population level analysis & evaluation, not individual intervention. Kendall provides an excellent resource on the implementation of such registries while taking into account alternative sources of data on IPV, the safety of victims, and the work-load on medical professionals, for example [35]. Until violence is accurately measured widely, programs will be limited in their evaluations, risking investment in ineffective interventions.

## Data Availability

Brazil’s Secretaria de Vigilância em Saúde provided SINAN (VIVA/SVS/MS) data. This can be requested through the system as described by the Health Ministry: https://antigo.saude.gov.br/saude-de-a-z/ouvidoria-do-sus. The following data bases are publicly available: Mortality (Homicides)—http://tabnet.datasus.gov.br/cgi/deftohtm.exe?sim/cnv/obt10br.def. Hospitalizations—http://www2.datasus.gov.br/DATASUS/index.php?area=0203&id=6926 then go to "Epidemolóticas e morbidade" then " Morbidade hospitalar do SUS". Bolsa Familia—https://dados.gov.br/dataset/bolsa-familia-misocial. Human Development Index—http://www.atlasbrasil.org.br/acervo/biblioteca. Municipal data—https://www.ibge.gov.br/estatisticas/sociais/saude/10586-pesquisa-de-informacoes-basicas-municipais.html?=&t=downloads. Finanças do Brasil: 2011–2012: http://www.tesouro.fazenda.gov.br/pt_PT/contas-anuais. 2013–2016: https://siconfi.tesouro.gov.br/siconfi/pages/public/consulta_finbra/finbra_list.jsf;jsessionid=fqxi8lemPFswaZaJuxnQta1r.node2.
